# Transactions of Wisconsin State Medical Society

**Published:** 1892-08

**Authors:** 


### Transactions of the Twenty-seventh Annual Session of the Homœopathic Medical Society of the State of Wisconsin, held at Milwaukee, May 26th–27th, 1891.

To judge by these transactions, our friends in Wisconsin had a very pleasant and profitable meeting.

There is a paper in these transactions from Dr. Eugene Storbe, Denver, Col., on "Homœopathic Literature." He first calls attention to the Homœopathic Medical Journals. One after the other is made to pass review before the mental vision of the critic. All of them fare well at his hands, with the exception of the *Journal of Homœopathics* and The Homœopathic Physician. "The doughty *Journal of Homœopathics* was for a year the sole theoretical mouth-piece of the entire profession. New York was the illustrious home of this literary freak. The periodical was a very peculiar one, in that it assumed the guise of an echo, which repeated the mystical and ethereal statements of a Finckean philosophy. It was a good journal—that is, it was good in this sense, it died young. The direct cause of its early demise, I am informed, was a literary marasmus, complicated by a diarrhœa of high-sounding terms. It reached out into the unattainable, dived down into the unfathomable, and never brought up practical facts. It seldom contained anything of consequence in its columns. Its philosophic aspirations paralyzed its literary usefulness, and it died full of hope that the medical world would suffer a cataclysm thereby. Its five-dollar subscriptions, which did not come in, would not admit of its further continuance, and it was placed in an early and timely grave. In the language of the Hiberno-Latin epitaph 'let it R. I. P.'"

Now, is not this nice? Written by a so-called homœopathist! Doctor, if I did not and could not harmonize with the Hahnemannian homœopathists, I should surely not abuse them.

"THE HOMŒOPATHIC PHYSICIAN is an alleged journal of our school and is published in the Quaker City. It is edited by a man named James. *We have never seen a copy of the work*, but from opinions we are able to glean of its character, is of the same order as the *Journal of Homœopathics*. It is engaged in admiring itself and abusing its neighbors."

The doctor has never seen a copy of THE HOMŒOPATHIC PHYSICIAN and never read a number, and still he claims that we are engaged in admiring ourselves and abusing our neighbors. What logic. No, doctor, we abuse nobody. We may criticize the practice of many pseudo-homœopaths, but abuse, no, never. According to the Lutheran classification of the ten commandments, the eighth commandment reads: "Thou shalt not bear false witness against thy neighbor." Doctor, procure Luther's Catechism and read the commentary.

